# Congenital Cytomegalovirus Infection in Pregnancy: Challenges in Early Diagnosis, Reinfection, and Secondary Prevention

**DOI:** 10.3390/v18070713

**Published:** 2026-06-28

**Authors:** Cinzia Auriti, Chiara Maddaloni, Sara Ronci, Alessandra Santisi, Ludovica Martini, Andrea Dotta, Maria Paola Ronchetti, Domenico Umberto De Rose

**Affiliations:** 1 Medicine Interdepartmental Faculty, Saint Camillus International University of Health and Medical Sciences, 00131 Rome, Italy; cinzia.auriti@gmail.com; 2 Neonatal Intensive Care Unit, “Bambino Gesù” Children’s Hospital IRCCS, 00165 Rome, Italy; chiara.maddaloni@opbg.net (C.M.); sara.ronci@opbg.net (S.R.); alessandra.santisi@opbg.net (A.S.); ludovica.martini@opbg.net (L.M.); andrea.dotta@opbg.net (A.D.); domenico.derose@opbg.net (D.U.D.R.)

**Keywords:** CMV, newborn, maternal serology, vertical transmission, placental infection, antiviral therapy, sensorineural hearing loss

## Abstract

Cytomegalovirus (CMV) remains one of the most relevant congenital and early-life infections in pediatrics because of its high global seroprevalence, lifelong latency, and potential for reactivation or reinfection. Biologically, the virus poses a particular threat during pregnancy, when maternal primary infection carries a substantially higher risk of transplacental transmission than non-primary infection, with fetal and neonatal consequences that vary according to gestational timing and host vulnerability. In children, CMV infection is common in the first years of life and may contribute to a broad spectrum of outcomes, ranging from asymptomatic infection to severe multisystem disease, neurodevelopmental impairment, and sensorineural hearing loss. Clinically, the document highlights the importance of timely maternal diagnosis, differentiation between primary and recurrent infection, and integration of prenatal, neonatal, radiological, and audiological assessment. Attention is given to symptomatic and asymptomatic newborns, preterm infants, and infants exposed through breast milk. The availability of antiviral strategies in pregnancy and infancy strengthens the rationale for early identification and risk stratification. Universal newborn screening emerges as a potentially valuable approach to improve case detection, enable prompt follow-up, and reduce long-term disability. Overall, a multidisciplinary and early-intervention framework is essential to optimize prevention, diagnosis, treatment, and long-term outcomes in pediatric CMV infections.

## 1. Introduction

Human cytomegalovirus (CMV) belongs to the Herpesviridae family and is classified as human herpesvirus 5, within the Betaherpesvirinae subfamily, alongside human herpesviruses 6A, 6B, and 7. Betaherpesviruses have a slower replication cycle than alphaherpesviruses and gammaherpesviruses. CMV, one of the largest human viruses, measures approximately 200 nm in diameter and is among the most widespread globally, with a seroprevalence exceeding 80% among women of reproductive age [1]. Congenital CMV infection is significant due to its global prevalence of about 0.64% and the potential for severe outcomes, with the probability of permanent sequelae in infected children reaching 17–20% [2,3]. CMV has an icosahedral structure, comprising four main components: an outer lipid envelope, a tegument, a nucleocapsid, and an inner nucleoprotein core containing the viral genome. The envelope consists of lipoproteins and at least 33 structural proteins involved in viral entry into host cells. The tegument contains structural proteins, including the pp65 antigen, an important target for diagnostic testing. The genome is a linear double-stranded DNA molecule measuring 64 nm, containing open reading frames that encode over 230 proteins. Among these is DNA polymerase, crucial for viral replication and a major target of all currently approved antiviral drugs [4].

The virus is acquired through contact at mucosal surfaces, blood transfusion, or organ transplantation. Cell-mediated viral spread begins after the initial replication phase. Human CMV infections can be classified as primary or non-primary. Congenital infection is acquired in utero, whereas neonatal infection may be perinatal or postnatal. CMV infection in childhood is also common during the paediatric age range. In immunocompetent hosts, primary CMV infection is usually asymptomatic, although it may present with fever, lymphadenopathy, and lymphocytosis, mimicking other viral illnesses. After primary infection, CMV establishes lifelong latency in a wide variety of cells, including leukocytes, endothelial cells, epithelial cells, and organs such as the salivary glands and kidneys. In these sites, the virus may persist and later disseminate through peripheral monocytes and circulating endothelial cells. During latency, active viral replication is undetectable, although residual transcriptional activity may persist at several viral gene loci, a phenomenon termed “sleepless latency” [5].

From this latent state, the virus may reactivate sporadically and spread mainly through prolonged viral shedding by asymptomatic seropositive hosts. Infections resulting from viral reactivation or reinfection with new strains are referred to as non-primary infections. Non-primary infections may be asymptomatic in immunocompetent individuals, but in immunocompromised hosts, they can lead to clinically severe disease. Congenital infection occurs when the virus is transmitted from an infected mother to the foetus through the placenta during pregnancy, causing foetal damage either directly or indirectly through inflammation at the maternal–foetal interface and consequent disruption of foetal development [6,7]. The risk of long-term sequelae is highest following maternal primary infection during the first trimester [8].

Furthermore, perinatal infection may occur when newborns acquire CMV during passage through the birth canal, whereas postnatal infection is acquired mainly through the breast milk of an infected mother. Early postnatal CMV infection is associated with increased morbidity, especially in preterm newborns [9].

## 2. Materials and Methods

This narrative review was conducted to summarize current evidence regarding congenital cytomegalovirus (cCMV) infection, with particular focus on maternal primary and non-primary infections, mechanisms of placental transmission, diagnostic strategies during pregnancy, prevention of maternal–fetal transmission, and neonatal management.

A literature search was performed using the PubMed/MEDLINE, Scopus, and Embase databases. Relevant articles published in English up to March 2025 were identified using combinations of the following keywords and Medical Subject Headings (MeSH): “cytomegalovirus”, “congenital cytomegalovirus”, “CMV pregnancy”, “maternal primary infection”, “non-primary infection”, “placenta”, “vertical transmission”, “prenatal diagnosis”, “IgG avidity”, “polymerase chain reaction”, “hyperimmune globulin”, “valaciclovir”, “newborn”, “congenital infection”, “antiviral treatment”, and “vaccine”.

Additional relevant publications were identified through manual screening of the reference lists of selected articles. Priority was given to systematic reviews, meta-analyses, randomized controlled trials, international guidelines, consensus statements, and landmark observational studies. More recent publications were preferentially included when addressing topics with evolving evidence.

As this manuscript was designed as a narrative review, a formal systematic review methodology was not applied. Consequently, study selection was based on relevance to the review objectives and the authors’ expert appraisal of the literature rather than predefined inclusion and exclusion criteria.

## 3. Results and Discussion

### 3.1. CMV Latency and Congenital Infection

Initial CMV infection triggers the production of specific IgM antibodies, followed by lifelong IgG antibodies. However, the mechanisms by which CMV establishes latency and persists within the host remain poorly understood. Latency plays a critical role during pregnancy, as, in seropositive hosts, which constitute a reservoir of viruses, episodes of reactivation and viral replication may happen, increasing the risk of fetal exposure and viral transmission. This risk becomes more significant as the seroprevalence of CMV rises in the population, potentially increasing the likelihood of fetal exposure, even in pregnant women who are already IgG positive [7]. CMV seropositivity during pregnancy is common, although its prevalence varies substantially across countries, being approximately 60% in France [10], 72% in Finland [11], and 98% in Brazil [12].

Consistent with these differences, the proportion of congenital CMV cases attributable to maternal non-primary infection is considerably higher in Brazil (90%), where maternal seroprevalence is very high, than in Finland (53%) or France (48%). This pattern differs from that observed for many other viral infections, in which maternal infection before pregnancy usually induces an immune response that provides substantial fetal protection [13].

In CMV infection, however, pre-existing maternal immunity does not reliably prevent viral reactivation, reinfection with a different strain, or transmission to the fetus. This distinctive pathophysiology, together with the incomplete protection conferred by the immune response, helps explain the difficulty of developing a truly effective vaccine. More recently, mRNA-based strategies have emerged as promising approaches for CMV immunization. In a rodent model, transamniotically delivered mRNA reached the fetal circulation through the placenta and induced a CMV-specific humoral immune response that persisted into the neonatal period, without apparent adverse effects on maternal, fetal, or neonatal morbidity and mortality. Although still experimental, this minimally invasive approach may represent a potential preventive option in the future [14].

### 3.2. CMV Infection in Pregnancy and the Role of Placenta

The probability of transplacental viral transmission to the fetus is a key clinical issue. Foetal infection in cases of early primary maternal infection occurs with an average incidence of 30% to 40% and increases in later pregnancy [15]. Immunological differences between mothers who transmit the virus and those who do not appear to play a role, although the exact mechanisms remain unclear. Maternal–fetal viral transmission is less common in immune women, occurring at a rate of 1% to 2%, indicating that some seropositive mothers can still transmit the virus. However, the literature supports that pre-existing adaptive immunity to CMV does not always prevent infection with a new CMV strain [16,17].

Early primary infection during pregnancy results in spontaneous abortion in approximately 15% of cases, typically with placental infection preceding fetal infection. Later in pregnancy, maternal CMV infection can cause preterm birth and intrauterine growth restriction in approximately 25% of affected newborns. These outcomes appear to be associated with placental pathology, suggesting that placental infection precedes viral transmission to the fetus [18].

The placenta protects the embryo and fetus through a complex structural and immunological network that blocks pathogens and contains antimicrobial factors [19]. In particular, the chorionic villi represent the first fully functional interface between the fetal and maternal circulations. During the second and third trimesters, the cytotrophoblast layer gradually regresses, leaving the syncytiotrophoblast as the primary barrier at the maternal–fetal interface. This layer facilitates the exchange of gases, nutrients, and waste products while also serving as a selective barrier against microbial invasion. Another key component of the placenta is its population of resident immune cells, known as Hofbauer cells, which are fetal-derived macrophages located within the chorionic villi. These cells perform essential immunological functions, including antigen presentation, phagocytosis, cytokine production, and coordination of both innate and adaptive immune responses [20]. Hofbauer cells are frequently targeted by various pathogens, including viruses, because of the expression of surface proteins such as CD4, which may facilitate viral entry. However, CD4 expression is believed to play a regulatory rather than pro-inflammatory role. Some studies also suggest that Hofbauer cells may contribute to the maternal-to-fetal transfer of antibodies via surface Fc receptors, potentially supporting neonatal immunity [21].

In the context of primary CMV infection during pregnancy, studies comparing women who transmit the infection to their fetus with those who do not have highlighted several immunological factors that may reduce the risk of transmission. These include the presence of neutralizing antibodies targeting CMV glycoprotein B, high-avidity IgG, and antibody-dependent, Fc-mediated cellular cytotoxicity, all of which appear to contribute to protection against fetal infection [22,23,24,25].

The group of Maidji and Pereira found that in women with a weak antibody response to CMV, IgG/virion immune complexes undergo transcytosis via neonatal FcR expressed by placental syncytium-trophoblasts. This process helps the virus avoid destruction by chorionic villi macrophages and spread to fetal vessels [26,27,28].

Thus, passive treatment with CMV hyperimmune IgG may be biologically plausible, although its efficacy remains controversial because it has not been confirmed in large randomized controlled trials [29]. Moreover, peripheral blood T-cell responses (CD4+, CD8+, and CD45RA+ memory cells) have been associated with lower rates of viral transmission to the fetus. Alfi et al. used an ex vivo model of CMV infection at the maternal–fetal interface to identify immune correlates of protection against transplacental transmission. Their findings indicate that CMV-specific, decidual tissue-resident memory CD8+ T cells are key drivers of protection. This protective response is absent in primary-infected tissues from CMV-seronegative women but is associated with resistance to non-primary infection in decidual tissues from HCMV-seropositive women [30].

However, these peripheral immune events may not fully reflect those occurring at the maternal–fetal interface, which could play a more significant role in determining the likelihood of fetal infection. The initial stage of placental infection probably occurs on the maternal side of the placenta, that is, in the maternal decidua, where invasive fetal cytotrophoblasts coexist with maternal immune cells. A study comparing placentas from women with primary or recurrent CMV infection found the virus mainly in fibroblasts and villous endothelial cells, as well as in macrophages, lymphocytes, and trophoblasts. Mothers of symptomatic infants showed more chronic villitis and greater villous alterations than mothers of asymptomatic infants. Additionally, mothers with non-primary infections had more damaged decidua than those with primary infections [31]. During pregnancy, chorionic villi are bathed in maternal blood, which comes into direct contact with the syncytiotrophoblast, the outer layer of the villi, and with cytotrophoblasts that invade the uterine vessels [31].

The hypothetical pathway of CMV fetal infection was demonstrated in a sophisticated experimental study conducted by Fisher et al. in the early 2000s. First-trimester chorionic villi and isolated cytotrophoblasts were exposed in vitro to CMV: syncytium-trophoblasts were not infected, while underlying cytotrophoblast clusters expressed viral proteins [32].

Cytotrophoblasts supported viral replication in vitro, impairing their differentiation and invasion of the uterine wall. Infection appeared to affect the overall cell population, as villi containing infected cytotrophoblasts showed altered architecture and reduced invasive capacity compared with uninfected villi. These findings suggest that CMV may reach the embryo–fetus through infection of underlying and invasive cytotrophoblasts. In addition, CMV impairs cytotrophoblast function, which may help explain the adverse pregnancy outcomes associated with maternal infection [33].

It is also relevant in the context of placental infection to consider the broad repertoire of CMV-encoded immune-modulatory genes that shape host antiviral responses at the maternal–fetal interface. CMV has evolved multiple gene products that interfere with antigen presentation and innate immune recognition, including viral homologues or functional mimics that modulate leukocyte activation, cytokine signaling, and natural killer (NK) cell surveillance. This is particularly important in the placenta, where decidual NK cells contribute to immune homeostasis, trophoblast invasion, vascular remodeling, and containment of infection. Several CMV immune-evasion mechanisms converge on NK-cell pathways, for example, by altering the expression or function of ligands involved in NK-cell activation and inhibition, thereby reducing the capacity of infected cells to be recognized and eliminated. In this setting, viral interference with NK-cell responses may facilitate local viral persistence, perturb placental immune balance, and potentially contribute to transplacental transmission and adverse fetal outcomes. Therefore, CMV genes targeting NK-cell immunity are of particular interest when discussing the impact of infection on placental health [34].

Infection in pregnancy and damage to the maternal–placental interface can lead to a hypoxic environment, resulting in compensatory placental remodeling. The balance between vascular endothelial growth factor (VEGF), which promotes angiogenesis, and placental growth factor (PlGF), which modulates trophoblast growth and differentiation, regulates normal placental development from early pregnancy onward. Under experimental hypoxic conditions, cultured cytotrophoblasts significantly upregulate VEGF expression and develop markedly dilated blood vessels around the villi to facilitate oxygen transfer to the fetus. This response suggests an inflammatory reaction aimed at increasing villous surface area to compensate for uteroplacental hypoxia. A similar phenomenon, also observed in women who smoke during pregnancy, may help explain the low birth weight of infants affected by congenital infection [35].

### 3.3. Which Women Are at High Risk of Transmitting the Infection to the Fetus?

All women are susceptible to CMV infection, and diagnosis during pregnancy can be challenging in the absence of prenatal serological screening, regardless of whether the infection is primary or recurrent. Clinical manifestations in pregnant women with CMV infection are often vague, minimal, or absent. Symptoms occur in approximately one-third of cases and typically resemble a mild flu-like syndrome, including fever or low-grade fever, malaise and myalgia. Consequently, diagnosis of CMV infection rarely relies on clinical presentation alone [36]. Laboratory findings are generally non-specific and may include mild lymphocytosis and elevated transaminases. Therefore, diagnosis relies primarily on virological and serological testing [37]. Detection of CMV DNA by polymerase chain reaction (PCR) in biological fluids such as saliva, urine, or blood represents a major diagnostic approach, with high sensitivity and specificity, particularly when performed within the first weeks after maternal infection or in neonatal screening contexts. However, diagnostic accuracy may vary depending on whether the maternal infection is primary or non-primary, as well as on the timing between infection and testing [18]. Furthermore, Tanimura et al. reported that the presence of fetal ultrasound abnormalities and a positive PCR result in uterine cervical secretions were independent predictive factors for congenital CMV infection in CMV-IgM-positive women [38]. Table 1 summarizes the serological profiles observed in women undergoing evaluation of their CMV infectious status.

### 3.4. Maternal Primary Infections (MPI), Serological and Molecular Tests

Primary infection is characterized by the absence of IgG, the presence of IgM, and variable PCR results. The highest risk of maternal–fetal CMV transmission and neonatal complications occurs during early primary infection, particularly in the periconceptional period and the first trimester. Chatzakis and Ville, in a large meta-analysis, found that first-trimester infections carry a 23% risk of late sequelae in neonates, compared with 0.1% and 0% when infection occurs in the second or third trimester, respectively [39]. Similarly, Faure-Bardon et al. studied 255 women with primary infection and their 260 infected children, analyzing follow-up data when the children were at least 1 year old. The timing of primary infection was determined by repeated measurements of maternal IgG and IgM, together with IgG avidity in serum collected during each trimester. Infection dating was prospective in 86% of cases and retrospective in 14%. Children were followed from birth to 48 months. At 24 months, rates of sensorineural hearing loss and/or neurological sequelae were 32.4% after first-trimester infection, 0% after second-trimester infection, and 0% after third-trimester infection (*p* < 0.0001). CMV infection appears to be more severe during the embryonic period, leading the authors to recommend auditory and neurological follow-up only for neonates with congenital infection after first-trimester maternal infection [40]. Therefore, CMV screening should ideally be performed before or at the beginning of pregnancy to assess maternal serological status and identify women at risk of primary infection during gestation. In women with negative CMV serology, retesting every 4 weeks until 14–16 weeks of gestation is advised, as primary maternal CMV infection is diagnosed by the presence of specific CMV IgM [2].

The latest-generation IgM assays have high sensitivity but lower specificity because IgM may persist after primary infection. If IgM is positive, IgG avidity testing is recommended to help confirm recent primary infection [41].

Conversely, current IgG avidity assays may help differentiate between recent (<3 months) and older (3–6 months) primary maternal infections. Consequently, high IgG avidity in the first trimester strongly argues against a primary infection acquired during that period, including the periconceptional period (±2 weeks from conception) and the preconceptional period (2–8 weeks before conception).

There are three key limitations of CMV IgG avidity testing:

1. Threshold variability: low- and high-avidity cutoffs vary across commercial assays;
2. Timing effect: test timing can critically influence negative predictive value; intermediate-to-high values after 21 weeks of pregnancy do not exclude primary infection, whereas high IgG avidity in the first trimester correctly identifies past infection;
3. Prolonged low avidity: unusually persistent low-avidity IgG (lasting more than 18 weeks) may lead to misdiagnosis of primary CMV infection, especially when CMV IgM is also detected.

Whenever preconception testing is performed, CMV serology should be recommended, and IgG avidity testing may be useful when recent primary infection is suspected.

In cases of recent primary infection, IgG avidity is often low (80–90% of cases) or intermediate. Notably, intermediate avidity can persist for up to six months after infection. When detected during suspected primary infection, retesting serum in specialized laboratories is recommended. Repeat testing can help clarify whether the infection is truly recent or has been present for a longer period, with important implications for clinical management [42,43].

Detection of CMV DNA in maternal blood may support the diagnosis of recent infection, particularly in the early phase, but its sensitivity is variable and DNAemia is transient. Viral DNA levels decline over time and may become undetectable, limiting their diagnostic value. Moreover, the presence of CMV DNA in blood or urine does not allow distinction between primary and non-primary infection nor accurate determination of the timing of infection, as viral shedding may persist for months with significant interindividual variability [44].

### 3.5. Maternal Non-Primary Infections (MNPI)

Diagnosing non-primary CMV infection without prior serological history is challenging [2]. No single laboratory test can reliably identify women with pre-existing immunity who are at risk of transmitting infection to the fetus, particularly in non-primary infections [45]. Serological markers are of limited value in non-primary infection, as CMV IgM is detected only in a minority of cases and may also persist or reappear during reactivation or reinfection, while increases in IgG titers are inconsistent and non-specific. Molecular testing is also limited, as CMV DNA may be detected intermittently in blood or other biological fluids, reflecting transient viremia and variable viral shedding. Thus, the diagnosis of non-primary CMV infection is often presumptive, and confirmation of fetal infection relies on invasive testing, particularly amniocentesis with CMV DNA detection, while serial serological and virological monitoring may provide only supportive information [8,46].

### 3.6. Secondary Prevention of Maternal–Fetal Infections

#### 3.6.1. Administration of Hyperimmune Immunoglobulins

Longstanding efforts have aimed to prevent mother-to-child transmission of CMV through intravenous administration of hyperimmune immunoglobulins to the mother [47]. The rationale for this approach is that immunoglobulins may act as a biological barrier to transplacental viral passage, like what occurs in most pregnant women with pre-existing immunity in whom infection reactivates without fetal transmission. For many years, targeted anti-CMV immunoglobulin therapy has therefore been offered to pregnant women as a form of secondary prevention. However, an Italian randomized controlled trial of 124 pregnant women found that monthly intravenous hyperimmune immunoglobulin at 100 IU/kg, started six weeks after infection onset, did not reduce congenital infection rates compared with placebo [48].

Studies such as that by Buxmann et al. [49] used monthly hyperimmune globulin administration based on an estimated terminal elimination half-life of 22.4 days for IgG antibodies [50]. Hamprecht et al. re-examined the pharmacokinetic profile of the CMV-specific antibody response in a pregnant volunteer with confirmed primary CMV infection who received intravenous hyperimmune globulin every four weeks. They observed periodic reductions in CMV IgG levels, with an estimated half-life of approximately 11 days, as well as variations in epitope-specific recombinant CMV IgG avidity and recurrent declines in epithelial-cell-specific neutralization capacity [51]. Although these findings may have influenced clinical outcomes, most obstetricians no longer recommend hyperimmune globulin for pregnant women with primary CMV infection.

In 2019, an observational study conducted at the University of Tübingen reported the effectiveness of biweekly administration of 200 IU/kg in pregnant women at a mean gestational age of 9.6 weeks (range 5.1–14.3) for preventing foetal CMV transmission in women with primary infection during the first trimester (by 14 weeks). The protocol required amniocentesis at least 6 weeks after infection onset, at 20 weeks of gestation. Initial results showed a 2.5% transmission rate (7.5% when cases with late-onset symptoms were included) in treated women versus 35.2% in controls, and all infected newborns were asymptomatic [29]. However, a 2021 double-blind multicentre study involving 206,082 pregnant women screened by IgM and IgG avidity, rather than documented seroconversion, found no difference in transmission rates between treated and untreated groups. Treated women experienced higher rates of adverse outcomes, including prematurity, low birth weight, and side effects such as severe allergic reactions, chills, and headaches. The trial was stopped for futility, and analysis of 394 participants showed no reduction in viral transmission or in the composite outcome of neonatal infection or death among women treated with hyperimmune immunoglobulins [52]. Debate on hyperimmune globulins continues, although most obstetricians do not currently recommend them [53].

#### 3.6.2. Prevention of Foetal Infection with Valaciclovir

In 2020, randomized and quasi-randomized studies evaluated oral valaciclovir at a dose of 8 g/day in pregnant women with primary CMV infection acquired during the periconceptional period or the first trimester. Valaciclovir is an antiviral prodrug with approximately 55% oral bioavailability, which is higher than that of acyclovir. It is used to treat herpesvirus infections such as HSV-1, HSV-2, herpes zoster, and varicella-zoster virus, and it may also help prevent CMV transmission. In the liver, valaciclovir is rapidly converted to acyclovir, which is then phosphorylated to acyclovir triphosphate. This active metabolite inhibits viral DNA polymerase, causing chain termination and blocking viral DNA synthesis.

The results of a meta-analysis showed that treatment with valaciclovir reduced vertical CMV transmission by 71%, with adjusted odds ratios indicating significant benefit. Three studies (n = 527 women) confirmed lower rates of neonatal infection and fewer pregnancy terminations prompted by severe foetal findings. Treatment was associated with a low rate (2.1%) of severe adverse effects, and earlier initiation correlated positively with better outcomes. Overall, high-dose valaciclovir effectively reduced CMV transmission with limited toxicity [54,55,56]. Oral valaciclovir at 8 g/day is recommended for women with primary maternal infection occurring during the periconceptional period or first trimester, starting as soon as possible after infection and continuing until amniocentesis at 20 weeks of gestation. To enable early treatment, prompt CMV serological testing in the first trimester is recommended, with repeat testing at 14–16 weeks for seronegative women. A real-life multicentre Italian observational study including 447 pregnant women (205 treated with valaciclovir and 242 untreated) confirmed that valaciclovir significantly reduces the rate of congenital CMV diagnosis at the time of amniocentesis, with a good tolerability profile, and showed that treatment is also associated with reduced rates of pregnancy termination and symptomatic congenital CMV infection at birth [2,57].

According to a meta-analysis, 21% of women experienced side effects such as nausea or headache. Mild to moderate acute renal failure occurred in 2% (three cases) and resolved after valaciclovir discontinuation [54].

Preventive therapy should be discontinued in women with a negative amniocentesis result. Women with confirmed foetal infection benefit from serial targeted foetal ultrasound examinations and third-trimester MRI, which provide complementary prognostic information [58].

### 3.7. Congenital Cytomegalovirus Infection in the Newborn

Approximately 1 in 200 neonates in high-income settings is affected by congenital CMV, making it the most common congenital infection [59]. Approximately 11–13% of infected infants are symptomatic at birth, and an estimated 40–58% of these infants will develop permanent sequelae. Even in the absence of neonatal symptoms, some infected children may later develop sequelae such as sensorineural hearing loss (SNHL), intellectual disability, and neurodevelopmental delay, which occur in approximately 8–15% of cases [60].

Congenital CMV infection should be suspected in a child born to a mother with documented infection (e.g., positive CMV PCR in amniotic fluid and seroconversion), suspected infection (e.g., flu-like syndrome during pregnancy or positive IgM results not further investigated), or prenatal ultrasound abnormalities suggestive of infection, such as ventriculomegaly, cerebral hyper echogenicity, subependymal cysts, hyperechogenic bowel, or hepatic calcifications [2].

In addition, any newborn presenting with symptoms, laboratory abnormalities, or ultrasound findings consistent with congenital CMV infection should be evaluated (Table 2) [2].

The gold standard for the diagnosis of congenital CMV infection is a positive real-time PCR result for CMV DNA in urine or saliva collected within the first 3 weeks of life. Comparison of PCR testing on saliva versus urine shows high sensitivity (93–100%) and negative predictive value (98–99%), together with moderate specificity (91–99.7%) and a relatively low positive predictive value (49–73%) [2]. False-positive saliva results may result from contamination by genital secretions or recent breastfeeding and are generally associated with low viral load. Therefore, a positive PCR result on saliva should be confirmed by PCR testing on urine [2]. After the first 3 weeks of life, the presence of CMV DNA in urine or saliva cannot definitively establish congenital infection. In children older than 3 weeks, routinely collected dried blood spots (DBS) from the first week of life can be examined retrospectively to detect CMV DNA in neonatal blood by PCR, thereby enabling diagnosis. However, the sensitivity of this method remains debated. According to a meta-analysis, PCR testing on dried blood spots (DBS) showed high specificity but variable sensitivity, with pooled estimates ranging from 84.4% to 99.9% [61]. Therefore, although a positive DBS PCR result is highly reliable for confirming congenital CMV infection, a negative result does not exclude the diagnosis. This limitation is mainly due to the lower viral load and smaller blood volume available in DBS samples compared with urine or saliva, which reduces the likelihood of detecting CMV DNA and makes DBS PCR less sensitive than testing performed on fresh neonatal urine or saliva collected within the first 3 weeks of life.

About 30% of infected neonates do not exhibit IgM at diagnosis, and IgM testing in neonates has a low sensitivity, making it unsuitable for diagnosing congenital CMV in neonates [62].

One of the major risk factors for long-term sequelae is MPI during the first trimester. A clinical virologist should assess serological findings to determine the timing of MPI as accurately as possible. The effects of MPI and maternal non-primary infection on long-term infant outcomes appear to be broadly comparable [45]. The clinical examination, laboratory testing, and specialized assessments required to determine whether congenital infection is symptomatic or asymptomatic are summarized in Table 3. Some of these findings at birth have variable prognostic value and should therefore be carefully considered when decisions about treatment and follow-up are made. Current management is increasingly focused on risk stratification and targeted follow-up. Children with CMV and confirmed transmission during the first trimester, or with unknown timing of transmission, should be followed for at least 6 years to ensure appropriate specialized management. By contrast, such prolonged follow-up may not be necessary in children born after documented MPI during the second or third trimester of pregnancy [2].

Microcephaly has high specificity for poor neurological outcome when compared with birth weight (i.e., HC z score − weight z score < −2). By contrast, in newborns without other symptoms, symmetric IUGR may not necessarily predict an adverse outcome [44].

When a newborn shows clinical signs of CMV infection at birth, sensorineural hearing loss, chorioretinitis, or cranial ultrasound abnormalities, brain MRI is recommended. MRI is also indicated in cases of maternal primary infection during the first trimester or when the timing of maternal infection is unknown. Normal neuroimaging predicts normal or near-normal neurodevelopmental outcomes, whereas significant abnormalities are associated with a poorer prognosis [63,64,65].

According to the recent European consensus (2024), antiviral therapy is recommended for symptomatic congenital CMV infection, particularly in cases with central nervous system involvement, microcephaly, or chorioretinitis (for 6 months), and in selected cases of isolated hepatitis or thrombocytopenia (for 6 weeks). By contrast, antiviral therapy is not recommended for asymptomatic infants or those with isolated intrauterine growth restriction [2].

Oral valganciclovir (16 mg/kg twice daily) is the treatment of choice, whereas intravenous ganciclovir is reserved for selected clinical situations. Therapy should be started as early as possible, ideally within the first month of life, and may be considered up to 12 weeks of age in selected cases [66].

Evidence regarding hearing outcomes is mixed: although early treatment may reduce hearing deterioration in some infants, initiation beyond the neonatal period does not appear to improve hearing, and prolonged therapy offers only modest long-term benefit. Careful monitoring is required because of potential haematological and hepatic adverse effects [66,67].

### 3.8. Vaccines and Future Perspectives

Although vaccine candidates aimed at reducing the risk of congenital CMV are advancing through clinical trials, it is likely to be several years before a licensed product becomes available. Until then, hygiene-based behavioral measures remain the only strategy currently available for primary prevention. The complex life cycle of CMV, its ability to evade host immunity, and the lack of clearly defined immune correlates of protection make vaccine development challenging [68].

Several vaccine platforms have been investigated, including live-attenuated, protein subunit, viral vector, DNA, and, more recently, mRNA vaccines, targeting key antigens such as glycoprotein B (gB) and the pentameric complex, as well as T-cell targets like pp65 [69]. Despite extensive research, several candidates have failed to demonstrate clinical efficacy in phase III trials [70]. Moderna® (Moderna, Inc., Cambridge, MA, USA) has developed an mRNA-1647 vaccine against CMV, but in October 2025, Moderna® announced that the Phase 3 mRNA-1647 vaccine candidate failed to meet its primary efficacy endpoint and its development was stopped [71].

Protective immunity is thought to involve both neutralizing antibodies against glycoprotein B and the pentameric complex, as well as cellular immune responses [69]. Vaccination of young children may provide indirect protection by reducing transmission to pregnant women [72].

However, the immunological deficits that allow reinfection in seropositive individuals, including pregnant women, remain unclear, and studies are urgently needed to define these deficits [73,74].

## 4. Conclusions

Congenital CMV infection remains one of the most challenging infections in maternal–fetal medicine because of its high global burden, its unpredictable transmission patterns, and the potentially severe long-term consequences for the child. Despite substantial progress in understanding viral pathogenesis and placental transmission, important diagnostic gaps remain, particularly in the identification and timing of maternal non-primary infections, for which no single serological or molecular marker is sufficiently reliable. Early recognition of primary maternal infection, especially in the periconceptional period and first trimester, is crucial because this is the setting associated with the highest risk of fetal damage and long-term sequelae.

Current evidence supports valaciclovir as the most promising strategy for reducing vertical transmission in selected women with primary infection acquired around conception or in early pregnancy, whereas the role of hyperimmune globulins remains controversial and is not supported by consistent evidence. After birth, prompt virological diagnosis and accurate stratification of symptomatic versus asymptomatic congenital infection are essential to guide neonatal evaluation, prognosis, and antiviral treatment.

Future progress will depend on three main advances: improved tools for diagnosing and timing maternal infection, especially reinfection; better prognostic markers linking maternal, placental, and fetal immune events; and the development of an effective vaccine. Until then, congenital CMV infection will remain a field in which early maternal identification, expert multidisciplinary assessment, and carefully targeted preventive strategies are the cornerstone of clinical management.

## Figures and Tables

**Table 1:** Diagnostic serological and molecular patterns in maternal CMV infection. The combination of CMV-specific IgG, IgM, and IgG avidity allows differentiation between primary and non-primary infection, while CMV DNA detection may support the diagnosis. However, no single marker is sufficient alone, and results must be interpreted in the clinical and temporal context.

CMV Maternal Infection Diagnosis			
	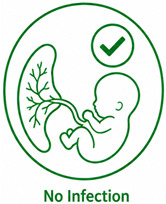	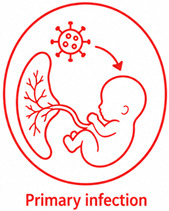	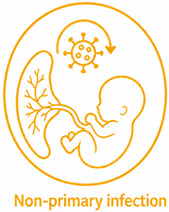
IgG	Negative	Negative	Positive
IgM	Negative	Positive	Positive/Negative
IgG avidity	-	Low	High
Viral DNA	Negative	Positive/Negative	Positive/Negative

**Table 2:** Clinical, laboratory and ultrasound findings attributable to congenital CMV infection.

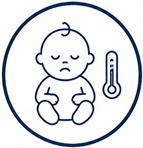 **Clinical Signs**	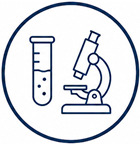 **Laboratory Abnormalities**	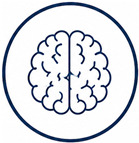 **Neurological Findings**	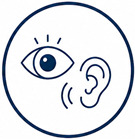 **Other Findings**
Microcephaly, intrauterine growth restriction (IUGR); Jaundice, hepatosplenomegaly, ascites; Petechiae-purpura, blueberry muffin rash; Abnormal neurological examination (lethargy, hypotonia, seizures, poor feeding)	Hyperbilirubinemia (conjugated bilirubin > 2 mg/dL); Hypertransaminasemia: alanine aminotransferase/aspartate aminotransferase (ALT/AST) at least 2 times the upper limit of normal; Anemia (according to reference hemoglobin and hematocrit values for age and sex); Leukopenia, isolated neutropenia (<1000/µL); Thrombocytopenia (platelet count < 100,000/µL)	Cranial ultrasound or magnetic resonance imaging (MRI) Inflammatory or destructive changes resulting from the direct effect of the virus or immune/inflammatory response: *-lenticulostriate vasculopathy,* *-germinolytic pseudocysts (caudothalamic, temporal, frontal),* *-occipital horn septations,* *-ventriculomegaly,* *-periventricular calcifications,* *-white matter abnormalities (i.e., increased signal intensity on T2-weighted MRI).* Brain developmental disruptions: *-cortical malformations (typically polymicrogyria or poorly developed sulcation),* *-cerebellar hypoplasia.*	Abnormal fundoscopic examination: Chorioretinal scarring Abnormal auditory brainstem responses (ABRs): sensorineural hearing loss (SNHL) (hearing threshold > 20 dB, unilateral or bilateral) Abdominal ultrasound: Ascites, hyperechoenicity of intestinal loops.

**Table 3:** Clinical examination, laboratory testing, and specialized evaluation needed at diagnosis.

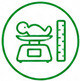	Weight, length and head circumference
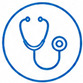	Physical and neurological examination
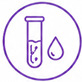	Full blood count, liver enzymes, bilirubin (total and conjugated)
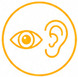	Ophthalmologic and audiologic assessment
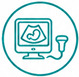	Cranial ultrasound
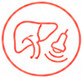	Abdominal ultrasound
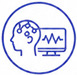	Electroencephalogram—EEG (if neurological impairment)
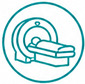	Brain MRI (possibly in the first month of life)

## Data Availability

No new data were created or analyzed in this study. Data sharing is not applicable to this article.

## Abbreviations

The following abbreviations are used in this manuscript:

ABRs	auditory brainstem responses
ALT	alanine aminotransferase
AST	aspartate aminotransferase
cCMV	congenital cytomegalovirus
CMV	cytomegalovirus
EEG	electroencephalogram
IUGR	intrauterine growth restriction
MPI	maternal primary infection
MNPI	maternal non-primary infection
MRI	magnetic resonance imaging
PCR	polymerase chain reaction
SNHL	sensorineural hearing loss
